# Healthier together. How arts on prescription can promote psychosocial wellbeing: a qualitative study

**DOI:** 10.1186/s12875-025-02800-6

**Published:** 2025-04-08

**Authors:** Anita Jensen, Annika Brorsson

**Affiliations:** 1 Competence Center for Primary Healthcare, Region Skåne & Social Medicine and Health Policy, Malmö, Lund University, Sweden and Nord University, Levanger, Norway; 2 Center for Primary Healthcare Research, Department of Clinical Sciences, Lund University, Malmö, Sweden

**Keywords:** Arts on prescription (AoP), Primary healthcare, Participatory arts, Mental health, Psychosocial wellbeing, Social prescribing, Social community

## Abstract

**Background:**

Mental health problems are an increasing challenge for primary healthcare. It puts strain on the healthcare professionals with limited time and resources. In several countries, healthcare professionals can refer patients with mental health issues to structured arts programmes, namely Arts on Prescription (AoP). This study explores the qualitative findings from a 3-year study on Arts on Prescription.

**Methods:**

Primary healthcare patients were recruited to participated in a 10-week group-based arts programme, twice a week for 2 h (22 sessions over 10 weeks, comprising a mixture of arts and cultural activities facilitated by arts professionals) referred from 18 different primary healthcare centres. Twenty-eight participants volunteered to be interviewed using a semi-structured one-to-one approach. The transcribed interviews were analysed using a thematic analysis.

**Results:**

Three themes were identified as Social community & Connectedness, Self-efficacy and Routine & Structure. Through the identified themes positive effects of psychosocial wellbeing is described as reported by the participants connecting to their experiences of participating in the Arts on Prescription programme including finding common grounds, feeling healthier and more human, connecting with inner resources, and establishing new routines.

**Conclusions:**

The findings highlight the potential of Arts on Prescription programmes to promote holistic psychosocial wellbeing, and to facilitate personal growth through community engagement and structured arts activities with salutogenic approaches.

**Supplementary Information:**

The online version contains supplementary material available at 10.1186/s12875-025-02800-6.

## Introduction

Arts on Prescription (AoP), also known as Arts on Referral (AoR), has gained increasing recognition in several countries as an approach to enhance social connectedness between individuals and communities through creative engagement. This initiative aims to improve overall health and wellbeing by fostering participation in arts and creative activities [1–3]. AoP operates within the broader framework of Social Prescribing (SP), a mechanism that enables primary care professionals, such as general practitioners and other healthcare providers, to refer patients to community-based initiatives. These initiatives encompass a range of activities, including gardening, cooking, communal walks, creative arts, physical exercise, and other group-based engagements [4, 5].

Many patients visiting primary healthcare have mental health or social problems that health professionals currently do not have sufficient resources and limited time to support [6]. Primary healthcare providers in Sweden have recognised and highlighted the need for more holistic approaches for people with mental health problems [7, 8]. They have perceived that AoP programmes are beneficial for patients in terms of motivation, creating routines, providing social interactions, and increasing self-esteem. Having complementary offers for patients with mental health problems and the possibility of referring patients to AoP in combination with conventional treatments was also appreciated [7]. Providing integrated care and patient-centred care (PCC) means putting people and communities (not diseases), at the centre of health systems and empowering people and communities to take charge of their health. PCC aims to make a functional life, affirming the ethical principles of respect for persons and equality, striving to make the health system more responsive to the health needs of patients [9].

The aim of the study was to investigate the effect of participation in a 10-weeks AoP programme for primary care patients with stress, anxiety, mild to moderate depression, or those experiencing loneliness/social isolation to consider AoP as a patient-centred intervention for primary healthcare in Sweden. The primary focus is to understand psychosocial effects of participating in the AoP programme and some of the processes leading to better psychosocial wellbeing.

This study aims to explore the psychosocial effects of participating in the AoP program, with a particular focus on the ingredients that contribute to improved psychosocial wellbeing. Specifically, it examines patients referred from primary healthcare, offering insights into how group-based arts activities can enhance health and wellbeing. These findings could inform the development of new strategies for practical implementation in Sweden. Additionally, the study seeks to address an underexplored area by providing insights to the potential of an alternative care pathway that is underpinned by a PCC approach.

This current paper presents some of the qualitative findings. Other parts on our study are presented in other publications, including a mixed methods study measuring wellbeing and salutogenic health and a study investigating the mechanisms in arts participation.

## Method

### Participants

The qualitative study included 28 participants (primary healthcare patients with anxiety, stress, depression, or those in risk of loneliness/social isolation) with the following gender identities: Women *n* = 21, Men *n* = 6 Other *n* = 1. The median age was 55 years old. In the group of interviewed participants, different backgrounds, gender, and age were represented reflecting the overall group of participants (total population *n* = 112).

### Referral process

Patients were referred to the study and the AoP programme by their primary healthcare provider (GP, nurse, psychologist, rehabilitation coordinator, etc.) after a consultation and clinical assessment of suitability for the study/AoP programme. Eighteen different primary healthcare centres in Malmö, Sweden referred patients to the study. After receiving the referral, an AoP-coordinator contacted the patient to provide additional information and an invitation to an introduction meeting.

### Inclusion criteria

The following participants were included in the study and the wider AoP programme: primary care patients, adults (18 years of age or above), mental disorders using the International Classification of Diseases 10th Revision (ICD-10-SE): stress (F43.1, F43.2, F43.8, F43.8 A, F43.8 W, F43.9) anxiety (F40, F40.1, F41.0, F41.1, F41.2, F41.9) mild to moderate depression (F32.0, F32.0, F32.1, F33) (indexes for common mental health disorders (CMD) in primary healthcare or risk of loneliness/social isolation assessed by the referring primary healthcare professional.

### Exclusion

Individuals under 18 years of age, who did not speak or read Swedish (interpreter not available) or with severe psychiatric conditions treated in psychiatric care.

### Ethics

The study has ethic approval from the Swedish Ethical Review Authority (Dnr 2021–02077). All participants were given comprehensive written and verbal information about the study and could withdraw from the study at any time, without questions asked. All participants gave informed consented to participation and publications. The research was conducted in accordance with the Declaration of Helsinki ethical principles for medical research. All participants are anonymised.

### Design of programme

The programme ran for 10 weeks with activities twice a week for two hours. Activities were scheduled the same weekdays during the whole programme to provide continuity. Nine different culture institutions collaborated by providing and facilitating arts and culture activities. In this way, no therapists were involved in delivering the activities. Table 1 lists the various arts and cultural institutions collaborating in the study and short description of the activities as well as the number of times that the participants visited the receptive participating organisations.

**Table 1:** The arts/culture institutions and activities included in programme

Venue	Activity	Number of times that participants visited
Malmö Opera	Music activities: listening to mini concert flowed by discussion	1
Malmö Kunsthall (gallery)	Guided tour of the exhibition and arts workshop	1
Malmö Stadsarkiv (city archives )	Discovering new places and learning more about Malmö’s history through a guided city walk	1
Malmö Live (music venue)	Introduction to the music venue, recording own song and trying instruments	3
Kollaborativet (preforming arts)	Interactive stage performance with focus a sensory experience	1
Malmö Bibliotek (library)	Shared reading method using short stories and poems	5
Malmö Museer (museums)	Guided tours of the castle, the technical museum, and the collections as well as creative workshops	5
Form/Design Centre (arts and craft centre)	Guided tour of the current exhibition and arts workshop	1
Malmö Konstmuseum (arts museum)	City walk with a focus on public art and arts workshop	2

The programme was designed to give the participants a range of different aesthetic and creative experiences to stimulate different senses (sight, hearing, touch) and to enhance engagement in.

a variety of practices with focus on aesthetic experiences. Activities were active (making, creating, doing) and receptive (listening, seeing, feeling) and included different arts disciplines i.e., literature, music, song, visual arts (including public art) and performance arts.

We ran 12 groups from September 2021 to May 2024. The groups consisted of up to 12 participants. Participants met 20 times to engage in arts and culture activities and participated in one introduction meeting and meeting at the end of programme meeting, 22 meetings in total.

### Data collection

A qualitative exploratory descriptive approach within an interpretive framework using one-to one semi-structured interviews explored the participants perspectives and experiences of the AoP programme. An interview guide was used (see Supplementary file 1). Twenty-eight semi-structured one-to one interviews were conducted. The first author conducted 26 interviews, and two interviews were conducted by a research assistant. The average length of the interviews were 38 min. The interviews were conducted between November 2021 and May 2024. The interviews were held within a week after the invention ended. Two to three participants from each group volunteered to be interviewed. Interviews were conducted in Swedish, recorded, and transcribed verbatim.

### Data analysis

Using a thematic analysis (Braun & Clark, 2021) [10], the process involved six distinct phases, namely: (1) *Familiarization with data*; (2) *Generating initial code*; (3) *Searching for themes*; (4) *Reviewing themes;* (5) *Defining and naming themes*; (6) *Producing the report.* To ensure rigour and trustworthiness both authors independently read each interview transcript and made notes of potential codes, and through discussions a list of codes was created (phase 1–2). The codes were then applied across the data set with detailed coding undertaken in discussion with both authors. The coded data was then searched for patterns to develop sub-themes which were then clustered and organised into the final themes by the authors (phases 3–5). In the final phase (phase 6) a draft was prepared by the first author making final adjustments, the second author commented and verified, and the final version was approved by both authors. Table 2 provides an overview of the key themes, sub-themes and coding as well as examples of quotations from the participants. In this article, we present three themes; namely Social community & Connectedness; Self-efficacy; Routines & Structures.

**Table 2:** Key themes, sub-themes, coding, and examples of quotes

Key theme	Sub-theme	Coding	Examples of quotes
*Social community & Connectedness*	Common ground	- Making friends - Diversity - Social connections	“*To feel a sense of community and…and some friends…that is something I have not felt nor had for a long time”* (KuR217).
	Healthier together	- Belonging - Feeling safe - Decrease in social isolation/loneliness	*“Breaking social isolation has been incredibly important to me”* (KuR218)
*Self-efficacy*	Inner resources	- Coping with challenges - Initial uncertainty	*“I can ride the bus; I have learned to find my way around Malmö* [the city] *and I am not afraid to go out”* (KuR132).
	Feeling human again	- Self-discovery/growth - Motivation - My voice	*“So*,* I planned. And then I could cope”* (KuR157).
*Routine & Structure*	Establishing new routines	- New habits - Having a schedule - Getting out of the house	*“I’ve been struggling…it has been a battle every morning to get up and go there but I felt that I got energy from people and stuff like that. I felt that if I just go there*,* I’ll feel a bit better that day*” (KuR206).
	Healthy activity	-Break from negative patterns -Looking forward	*“[…] otherwise*,* I wouldn’t get a break from my bad moods. It was very good for me to have something in a schedule”* (KuR161).

## Results

The qualitative findings are presented as the following three themes: *Social community & Connectedness; Self-efficacy; Routine & Structure*.

### Social community and connectedness

All the participants reported that social interactions and community had had a positive impact on them. By (re)connecting with the world around them, the participants found strength and support in relating to others who understood and shared their experiences. As a participant described: “*to feel a sense of community and…and some friends…that is something I have not felt nor had for a long time”* (KuR217).

Participants also described how they experience resonance with others “*I found a lot of camaraderie with them [the group]…which I hadn’t yet found in my old circle of friends”* (KuR205) indicating a special connection and an ability to resonate with others in the group.

For others, the social community in the group had helped break social isolation” *I have been on sick leave for a long time and lost all my friends. I have one friend left* (KuR217) and *“breaking social isolation has been incredibly important to me”* (KuR218) highlighting the importance of social engagement to prevent isolation and increase opportunity for social connections. In addition, the group -based engagement with the arts activities were considered meaningful: *“I feel that I’ve done something meaningful during this period. I maybe had a bit of trouble with that before“*(179 KuR).

Some participants commented on how they valued the diversity within the group. *“It’s been a nice group*,* nice people. A very diverse collection*,* different ages*,* but the more you meet the more comfortable you get”* (KuR179) and “…*it’s fun with a wide age range because you get…many different references*” (KuR181). Common grounds were found, despite diversity: “*I felt we had a lot in common even though we were different*” (KuR102). Other participants commented on being on how being in the same boat made bonding with others easier.

The group dynamics within the groups appeared to have significance and the participants noticed the fluctuations in attendance of other participants. A participant commented: *“if there was someone missing you reflected on it”* (KuR125). Suggesting that a level of concern for other group members was present.

The safe environment within the groups enabled positive feelings and a sense of belonging. As a participant stated: “*You really feel that you are one of the group which I think felt good”* (KuR125) and another commented: “*I felt safe in this group. I could sort of open up”* (KuR127). While some liked the group sizes: “*I think it has been great that it has been a small group* (KuR199), there were also disappointment about other group members’ level of commitment, as a participant expressed: *“So we became three*,* four in the group…and one time it was only me. And I thought that was sad”* (KuR189).

Many participants reported that they were continuing to stay in touch with one or more people from the group. Some groups had planned activities together after the AoP programme including going to an art museum, going for a walk, and having coffee together as well as setting up a Facebook group.

Providing a safe environment, the social community in the groups developed friendships and have fostered meaningful relations enabling a sense of belonging among the participants. This feeling of resonance with others has helped break the social isolation and encouraged social engagement.

### Self-efficacy

Participants reflected on personal growth and self-discovery in terms adopting new behaviours that lead to specific actions e.g., connecting with others and their social environment*” I think*,* I have become better at relating to others*,* actually”* (KuR102). Others reported an ability to exert a level of control over their own motivation “*For me it was really an opening…to cultivate my brain and to be stimulated by something else”* (KuR111) and to find new interest: *“It opens your eyes to different things you never thought you could be interested in”* (KuR105). For another participant it was about the overall effect and regaining old interests: *Yes*,* on the whole*,* uplifting… uplifting effect and that I was taken out of my isolation*,* temporarily anyway. But also*,

*that I have been inspired to resume my previous activities as well. Writing and…and painting* (KuR 118).

The AoP-programme provided opportunity for participants to overcome challenges, and to train common undertakings. Some reported difficulties in perform everyday activities, such as using public transport and thereby increased self-reliance during the programme. As a participant stated: *“I can ride the bus; I have learned to find my way around Malmö* [the city] *and I am not afraid to go out”* (KuR132). Another participant considered different coping strategies for getting to the activities and that the AoP programme had provided opportunity to test new ways of coping and found that planning was helpful as she stated: “*So*,* I planned. And then I could cope”* (KuR157).

Some participants reported that they had initial anxieties and reluctance of participation in the programme. Anxiety is prevalent among patients with depression and often symptoms coexist [11] and it can be difficult to find strength and motivation to try something new, especially as many patients had already tried other care pathways. As stated by a patient: *“It was a bit scary at first*,* it was. But afterwards if felt much easier and you realise more people are in the same situation or similar situations”* (KuR105). Exposure to other peoples’ situations helped normalise feelings and emotions of participants’ own circumstances and experiences: *“Here I have felt normal and not inhibited in any way.…yes*,* together with like-minded” (*KuR217*).* Most patients mentioned the importance of being with people who had similar experiences and not needing to explain themselves.

Some participants reported that they had found self-acceptance and experienced personal growth. Through rediscovering a sense of identity and purpose, a participant states that *“I feel a bit like I’m becoming human again and this is how life should be*,* to feel like a human-being”* (KuR103). Another participant emphasised the significance of being part of something and to find ways to regain lost parts of oneself and to find joy after a long period of illness: *“It is important for me to have an interest and identity outside my illness. Therefore*,* it has been important for me to engage in arts activities*,* creative activities*,* and hobbies that I find joyful*” (KuR218). The programme provided a structured opportunity for participants to rediscover old interests and find new ones, which is vital for fostering a sense of purpose and fulfilment.

A participant expressed anxiety and sadness about the end of the programme– transition from the programme created uncertainty: *“Yeah…well*,* it feels like that. So*,* I want to go back*,* but I don’t really want to go back to that [previous job]. But…I hope it will work out and some other opportunities will come”* (KuR112). Others commented on AoP leaving an “empty space” and that they did not have follow-up appointments with their primary healthcare professional who referred them to the programme.

The positive experiences have increased coping mechanisms and self-efficacy. As stated by a participant: *“The various cultural and creative activities have given me so much joy and have meant so much to me*,* both in terms of my self-confidence and my emotional wellbeing”* (KuR218), linking arts participation to enhanced emotional coping.

By some level of self-accept and belief in a capacity to do things, feeling motivation anew and have control over own behaviour- the participants reported that they developed ways of relating to themselves, to others and to their community. But also, reluctance how they would cope after the programme not knowing if the newly found motivation and routines are so strongly embedded that they last.

### Routine and structure

Many participants emphasised the importance of having routines and structures in their lives, particular to maintain mental health wellbeing and more often such routines and structures had become limited due to ill-health or isolation.

Participants commented on how the AoP programme had supported them in getting new routines, as a participant reflected: *“I know that Tuesdays and Thursdays*,* I will go to different activities”* (KuR 103). Being part of the programme meant participating in different activities every Tuesday and Thursday for ten weeks at the same time. However, for some, this new routine was experienced as struggle: *“I’ve been struggling…it has been a battle every morning to get up and go there but I felt that I got energy from people and stuff like that. I felt that if I just go there*,* I’ll feel a bit better that day*” (KuR206). However, engagement in the arts and culture activities also created a new context for the participants where it was possible to be free and have a break from circles of negative thought. As a participant stated: *“The days at AoP*,* I’ve been able to let it go [negative thoughts] and kind of think about the here and now”* (KuR125).

Some participants had multiple health-related difficulties and therefore found participating challenging, however, they also understood that overcoming such challenges would make them feel better, as expressed by a participant: *“I slept so badly for a long time that I woke up five*,* six times because I was in pain and… but it was very positive that it also gave me some structure to my life”* (KuR158). The participant expresses finding a balance between the pain (caused by being active) and the benefit of the structures that the AoP programme provided.

One participant commented how participation and following a scheduled programme provided a break from circles of negative moods– even if it was only momentarily: *“I felt a little better when I was with the group but when I came home*,* I became…yes…worse again but I thought it was still better that I could get out of my house otherwise I wouldn’t get a break from my bad moods. It was very good for me to have something in a schedule”* (KuR161). Participation in AoP was considered a positive activity and meant that the participant left the house. Behavioural activation encourages people to engage in antidepressant behaviour such as maintain social connections and involve themselves in meaningful activities [12]. However, uncertainty about the long-term effect of the programme was raised by a participant: “*The days that I have been on AoP have been a bit happier and sometimes much happier. But in the long run*,* I don’t know*,* it’s hard to say…I would probably need to try eight weeks without AoP*,* then we will see if I can handle it”* (KuR206). The participant found it difficult to imagine what their days would be like without the scheduled activities.

With the support structures of the AoP programme, participants overcame challenges and while the achievement that meant a lot to the participants it also helped break negative routines of staying home. Establishing routines and structures can significantly benefit individuals experiencing feelings of hopelessness, and social isolation, and completing small tasks within a routine fosters a sense of accomplishment and progress, thereby counteracting negative self-perceptions [13]. Routines also introduce predictability and stability, providing comfort in situations where ill-health and social isolation render the world overwhelming. This predictability helps individuals regain a sense of control over their lives, which is particularly empowering during periods of battling with ill-health. Additionally, routines facilitate the setting and achievement of small, manageable goals, creating a pathway towards larger, long-term objectives.

## Discussion

In this discussion, we explore how social community and participating in meaningful group-based structured arts activities can support the psychosocial wellbeing of primary healthcare patients with mental health disorders or individuals who are socially isolated. The findings illustrate the multifaceted effects of participating in an AoP programme and the importance of addressing various aspects of health and wellbeing for this patient group.

In terms of making connections and feeling a sense of connectedness, the findings from this study align well with existing research on AoP which emphasises the importance of social connections and community in promoting mental wellbeing [14]. Previous studies have shown that social interactions and community involvement are critical for mental health wellbeing and recovery [15, 16]. The concept of “social prescribing,” where healthcare providers refer patients to community-based activities, including arts and culture, has been gaining traction as an effective approach to support mental health [17, 18]. The AoP programme’s emphasis on creating a supportive social community echoes the findings of these studies and demonstrate how connecting with people with similar experiences can considerably reduce feelings of isolation and improve overall mental health. Group-based arts interventions have been shown to have social and psychological benefits [14, 19]. The AoP programme’s strength lies in its ability to create a robust social community that addresses the pervasive issue of social isolation among patients with mental health issues. The importance of community engagement in mental health is important for promoting a sense of belonging, emotional support, and a network of like-minded individuals [20]. For many patients, long-term mental health issues lead to a significant withdrawal from social activities and a subsequent feeling of isolation. Creating safe environment in the AoP groups is instrumental in allowing participants to express themselves without fear of judgment. A feeling of safety and acceptance is important for fostering a sense of belonging and facilitating meaningful social and arts engagement, breaking cycles of social isolation and building new, supportive networks creating social connection and connectedness [21].

### A catalyst to improve self-efficacy

The enhancement of self-efficacy reported by the participants is supported by substantial evidence in the arts and health literature [19]. Engagement in the structured activities was found to increase self-esteem and confidence, providing individuals with a sense of achievement and enhanced control over their lives. The impact on participants’ self-efficacy mirrors the findings of studies that emphasize how arts-based interventions can empower individuals, helping them develop new skills and gain confidence in their abilities to manage their mental health [22]. According to salutogenic theory it is vital for positive health to be able find ways of developing skills and understanding for what is good for our health [23]. Many participants reported personal growth and a newfound confidence in their abilities to interact with others and engage in social activities. This shift in self-perception is crucial for mental health wellbeing, as it empowers individuals to take control of their lives and make positive changes.

An increased ability to cultivate interests and motivation was also reported leading to a positive impact on participants overall mental health. Engaging in arts and culture activities that stimulate minds and foster personal interests helps in combating the feelings of hopelessness and lack of motivation. Exposure to others’ situations within the group helped normalize participants’ feelings and emotions, making them feel less isolated and more understood. The shared experience fostered a sense of self-acceptance and personal growth, enabling participants to rediscover a sense of identity and purpose as well as feeling motivated to try new things (engage with education, re-entering the job market, take courses, interact with the social community). In this way, the AoP programme can be a catalyst for moving on in life and improve self-efficacy.

### Behavioural activation

Structured activities provide predictability and stability, which are important for individuals dealing with mental health issues. The positive effects of having a scheduled program, as experienced by AoP participants, resonate with findings that emphasise the importance of routines in maintaining mental health wellbeing (e.g., sleeping patterns, see Lyall et al., 2018 [24]). Behavioural activation, which involves encouraging individuals to engage more frequently in positive and meaningful activities that provide intrinsic enjoyment or a sense of accomplishment that helps to improve mood and alleviate symptoms of depression, is a recognised therapeutic approach in mental health treatment showing clinical effectiveness [25] and aligns with evidence-based practices that advocate for structured engagement as a means to improve mental health [26]. The AoP programme’s structured schedule helped participants overcome inertia and engage in activities, developing new routines, breaking negative cycles, and fostering a sense of accomplishment which resonate with previous research (e.g. see Dingle et al., 2013 [27]).Participants frequently highlighted the importance of having a structured schedule as well as noting that structured activities offered by the programme also serve as a distraction from negative thoughts. Further, overcoming the challenge of participating in scheduled activities despite physical or mental health difficulties gave participants a sense of achievement. Accomplishments are essential for counteracting negative self-perceptions and fostering a positive self-image [28]. Behavioural activation helps building an upward spiral of motivation and energy.

### Strengths and limitations

This study has several strengths and limitations. Two or three participants in each group volunteered to be interviewed. These participants attended the groups regularly, and thus they could share rich first-hand experience. The relatively large number of interviews also allowed data saturation, which means that no new themes were identified after the analysis of approximately 20 interviews. Other participants may have had other experiences, but since the purpose of this qualitative.

part of the study was to deepen the understanding of their experience; we believe that that aspect is satisfied by the quantitative data. This also means that the findings cannot be generalised but can be applied to similar contexts and the interviews were voluntary and therefore selection bias may occur.

The study has been conducted by two researchers; the first author is a social scientist, and the second author is a specialist in family medicine. This we believe, reduces the risk that preconceptions influence the findings.

## Conclusion

In conclusion, AoP programme has demonstrated significant potential in supporting the psychosocial health and wellbeing of primary healthcare patients dealing with mental health challenges or social isolation. By fostering social connections and a sense of community, the programme addresses the critical issue of isolation, promoting emotional support and a sense of belonging. Participants experienced increased self-efficacy, gaining confidence and control over their lives through engagement in scheduled arts-based activities. The structured nature of the programme also played a key role in promoting behavioural activation, helping participants develop positive routines and counteract negative self-perceptions. Finally, the AoP programme serves as a powerful catalyst for personal growth, social connection, and improved mental health, providing a holistic approach to addressing the complex needs of this patient group– and may reduce demands on primary healthcare professionals’ resources dealing with issues that are non-clinical– by using PCC salutogenic approach.

## Electronic supplementary material

Below is the link to the electronic supplementary material.

Supplementary Material 1

## Data Availability

The datasets generated and/or analysed during the current study are not publicly available but are available from the corresponding author on reasonable request.
