# Supplementary material for: Healthier together. How arts on prescription can promote psychosocial wellbeing: a qualitative study
Source: BMC Prim Care. 2025 Apr 8;26:100. doi: 10.1186/s12875-025-02800-6 (PMC11978017; doi:10.1186/s12875-025-02800-6)
Supplement: Supplementary file 1 — Supplementary Material 1 [file 12875_2025_2800_MOESM1_ESM.docx]

# **Supplementary file 1**

# Interview guide for Arts on Prescription research study 2021-2024.

## 1.Background information

- Have you been referred to other activities from your primary health provider (e.g. mindfulness, medical yoga, CBT)?

- Are you on sick leave? If yes, how long have you been on sick leave?

- How many times have you visited the GP surgery in the last year?

## 2.Reason for participation

- Who referred you to the programme?

- What is the reason for your participation in Arts on Prescription?

- What did you think when you first heard about the programme?

- What do you think about the primary healthcare offering cultural activities? (Examples of other activities in the community such as gardening rehabilitation, walking, handicrafts etc.)

## 3. Well-being

- What effect do you think participation has had on your health and well-being?

- Is there anything in particular that you think has changed?

- In what way has participation been beneficial/not beneficial for you?

## 4.Community

- What did you think of the group?

- Can you say something about the social relationships in the group?

- Will you keep in touch with anyone from the group? If yes, how? If no, why not?

## 5. Other

- If you had not participated in the Arts on Prescription programme, what would you have done?

- What are your plans now?

- In your words, what impact has Arts on Prescription had on you?
